# The Community Health Worker (CHW) Common Indicators Project: Engaging CHWs in Measurement to Sustain the Profession

**DOI:** 10.3389/fpubh.2021.674858

**Published:** 2021-06-22

**Authors:** Keara Rodela, Noelle Wiggins, Kenneth Maes, Teresa Campos-Dominguez, Victoria Adewumi, Pennie Jewell, Susan Mayfield-Johnson

**Affiliations:** ^1^Immigrant and Refugee Community Organization, Portland, OR, United States; ^2^Wiggins Health Consulting LLC, Portland, OR, United States; ^3^Director, Applied Anthropology Graduate Program, School of Language, Culture and Society, Oregon State University, Corvallis, OR, United States; ^4^Multnomah County Health Department, Portland, OR, United States; ^5^Manchester City Health Department, Manchester, NH, United States; ^6^Nottawaseppi Huron Band of the Potawatomi, Fulton, MI, United States; ^7^School of Health Professions, University of Southern Mississippi, Hattiesburg, MS, United States

**Keywords:** community health workers, measurement, popular education, participatory evaluation, community based participatory research

## Abstract

Despite progress in documenting the outcomes of Community Health Worker interventions, the lack of standardized measures to assess CHW practice has made it difficult for programs to conduct reliable evaluations, and impossible to aggregate data across programs and regions, impeding commitment to sustainable, long-term financing of CHW programs. In addition, while CHWs have sometimes been involved as data collectors, they have seldom been engaged as full partners in all stages of evaluation and research. This manuscript details the current work being done by the CI Project, demonstrating how CHWs are able to contribute to the integrity, sustainability, and viability of CHW programs through the collaborative development and adoption of a set of common process and outcome constructs and indicators for CHW practice and CHW program implementation.

## Introduction

Measurement is inherently political. Who has the power and money to measure; what is measured and who chooses; how measurement occurs and who decides; whether or not measurement is a requirement for funding; how and by whom data are collected, collated, analyzed, interpreted and presented—all of these questions reflect ideologies and relationships to power. As scholars in the movement to decolonize research and practitioners of various strands of participatory research have made clear (1, 2), what were previously thought of (by those in power) as value-free, objective decisions, are anything but.

This is particularly true in the case of measurement and evaluation in the Community Health Worker (CHW) profession. CHWs are trusted community members who work with others in their community and use a range of approaches to improve health and equity (3). As predominantly members of marginalized communities where health inequities are greatest, CHWs (who use titles including Community Health Representatives in Native/American Indian communities and Promotores/as in Latinx communities) experience the same oppression and denial of power experienced by their broader communities. Historically and still today, this has included the denial of power to identify research and evaluation questions, and design, conduct, and disseminate research and evaluation studies.

While CHWs have sometimes been involved as data collectors, they have seldom been engaged as full partners in all stages of evaluation and research, from conceptualization to analysis to publication. A recent systematic review of CHW research found that only 23 articles out of 130 included CHW participation in five or more intervention research phases. Across the phases of research, 98.5% of studies employed CHWs to implement the health intervention. CHWs were frequently involved in participant eligibility screening and/or recruitment (57.6%) and data collection (49.2%). CHWs were much less frequently involved in identifying the research question (10.8%), data analysis (2.3%), and research dissemination/action (10.8%) (4).

Based on the authors' collective experience, because of CHWs' strong connections with community members, the data they collect are often more accurate and extensive than data collected by non-CHWs. In the context of the pandemic, for example, CHWs have been able to learn more about possible sources of transmission than epidemiologists who lack the same levels of trust and connection.

Consequences of alienation from the knowledge production process for CHWs have mirrored consequences for people in other marginalized communities and include characterization through a white/academic/bureaucratic/colonizer/medical gaze (1, 2). Additionally, CHW studies and evaluations have often lacked the crucial perspectives of those closest to and most informed about the work, which has led in turn to the use of process measures that do not adequately capture the contributions of CHWs, and outcome measures that do not emphasize the outcomes CHW are uniquely able to achieve (5).

Another outcome of CHWs' and their communities' social location is chronic underfunding, including underfunding of research and evaluation. One of the consequences of underfunding has been an inability to use common measures to conduct longitudinal studies such as those which have been conducted in fields like nursing (6). Despite progress in documenting the outcomes of interventions led by CHWs (7–12), a lack of standardized measures to evaluate CHW programs and policies has made it impossible to aggregate data across programs and regions, impeding commitment to sustainable, long-term financing of CHW programs and positions. Aggregated data could also facilitate inferences about which aspects of CHW practice and program inputs lead to improved outcomes. Lack of comprehensive and easy-to-use indicators hampers the ability of many community-based programs to reliably report outcomes to funders. Lack of attention to the processes by which CHWs achieve outcomes has made it difficult to conclusively demonstrate the importance and effectiveness of particular CHW roles, skills, and qualities, and identify the kinds of support that programs need to provide to CHWs (5).

To address these issues, building on work conducted by the Michigan CHW Alliance, in 2015 CHWs and non-CHW researcher collaborators from five states formed the national CHW Common Indicators (CI) Project. The purpose of the CI Project is to contribute to the integrity, sustainability, and viability of CHW programs through the collaborative development and adoption of a set of common process and outcome constructs and indicators for CHW practice. Since its organizing Summit in 2015, CHWs have been at the forefront of the CI Project. Five of 16 attendees at the organizing Summit were CHWs, three of whom co-facilitated the Summit. CHWs have been actively involved in presenting about the project, participating on the project Leadership Team and Advisory Group, and publishing blogs and peer-reviewed journal articles about the project (13–15).

Between 2015 and 2019, the CI Project achieved several important goals, including engaging more than 100 CHWs, researchers and others from around the country who are committed to identifying and implementing common indicators through a participatory process (13), and compiling a robust set of 20 process and outcome constructs (see Table 1). Based partly on a strong track record of accomplishments, in 2019 the CI Project received an initial year of funding from the Centers for Disease Control and Prevention (CDC) via the National Association of Chronic Disease Directors (NACDD)^1^.

**Table 1:** Full list of recommended constructs with definitions.

**Process constructs**	**Definitions**
CHWs' job satisfaction	The extent to which CHWs are satisfied with their overall job conditions.
CHWs' compensation, benefits and promotion	The salary paid to CHWs in relation to their FTE and local cost of living, in addition to the presence or absence of health insurance, retirement, disability, and paid leave within their benefit package. Opportunities for advancement/promotion are also part of this construct.
Acceptance/Value of CHWs to the organization	The extent to which CHW work is considered a regular and valuable component of the employing organization's services.
Supportive and reflective CHW supervision	The extent to which CHWs feel they receive supervision from clinical and non-clinical supervisors that is supportive, reflective, and trauma-informed, not disciplinary and paternalistic.
CHW enactment of the 10 core roles	How often (in the past week, month, or year) individual CHWs or a group of CHWs within a program or organization enacted or engaged in each of the 10 core roles defined by the CHW Core Consensus (C3) project.
Participants' trust/satisfaction with CHW relationship	The extent to which participants feel they can trust the CHW(s) with whom they work, including trusting that a CHW will keep their private information confidential, and that a CHW is genuinely dedicated to their care and well-being. Also, the extent to which participants are satisfied with their relationship with their CHW(s), in terms of feeling genuinely respected and understood by their CHW(s).
CHW-facilitated referrals	Completed referrals facilitated by the CHW, through which the participant successfully receives attention, care, and/or resources from a clinic, other healthcare or social service agency or public service. CHWs will not be held responsible when necessary services are not available.
CHWs' involvement in policy making	The extent to which a CHW is able to be involved in policy making both within their own organization and in the larger community on work time and/or as part of their volunteer commitment.
CHW integration onto teams	The extent to which CHWs are members of a collaborative and communicative “team” with other providers within a clinic, school, social service agency, etc.
Use of popular/people's education in CHW training	The extent to which CHW training is informed by popular/people's education, which values, draws out and builds on what CHWs know through life experience.
**Outcome constructs**	**Definitions**
Participant self-reported health status	A participant's own assessment of their physical, mental, and emotional health.
Participant quality of life	A participant's perception of their position in life in the context of the culture and value systems in which they live and in relation to their goals, expectations, standards and concerns (WHO).
Participant health and social needs	Health and social needs currently experienced by the participant, e.g., food, transportation, water, and housing insecurity.
Participant knowledge, attitudes and behaviors	A participant's knowledge, attitudes and behaviors related to specific health conditions.
Participant social support	The level of support (i.e., assistance/help) that participants perceive from others to deal with regular and emergent life challenges, including economic, social, health, and emotional challenges.
Participant empowerment	A composite measure assessing both actual and perceived empowerment. Includes the following domains: self-efficacy, sense of community, perceived control at the community level, decision-making ability, education/knowledge/skills, critical consciousness, optimism, inner peace, communication, resources.
Participant cost of care	The total cost of a participant's health care in a given period of time, with a focus on high cost emergency services.
Participant utilization of health services	A participant's use of health services in a given period of time, for example, use of emergency vs. routine primary care services.
Participant health outcomes	A participant's physical, mental and/or emotional health status, as assessed by a clinician.
Policy and system change	Policies and system changes that address CHW workforce development and sustainability as well as policies that promote population health and address inequities (i.e., many different policies at multiple levels of government, business, etc.).

The purpose of the CDC-funded 2019-2020 scope of work was the collaborative selection of 10 priority constructs (from the list of 20 in Table 1) and development of associated indicators for evaluation of programs, systems, and investments involving CHWs. If adopted, these indicators will illuminate (1) the processes by which CHWs achieve positive outcomes at multiple levels (individual, community, and system), (2) the outcomes themselves, and (3) the key kinds of support that CHWs need to be successful, across programs and diseases or conditions. In addition, there are currently few specific indicators available to measure process and outcome constructs across CDC CHW programs and initiatives. The CI Project's 2019–2020 work addressed this gap, thus strengthening the evidence regarding CHW contributions to improving health and reducing inequities. The project did not require IRB approval as it did not include research participants.

This article describes how Project leaders were able to enhance the engagement of CHWs and achieve project objectives during the first year of CDC funding and in the midst of the COVID-19 pandemic through a series of activities that were based in popular education methodology. The article then discusses findings and lessons learned. After exploring conceptual and methodological constraints of the project, the manuscript concludes with a summary of next steps and some persistent questions posed by a project of this type.

## Context

The setting for the CI Project is the CHW field in the United States. Organizational settings include community health centers; community-based organizations; academic health centers; universities; health plans; state, local, and national CHW associations; and a range of other organizations that are led by and employ CHWs. Principal actors (the population) are CHWs, who by definition are members of the communities they serve. Other stakeholders and constituents include university- and community-based researchers and evaluators; CHWs' colleagues in their places of work; program administrators; CHW supervisors; and those involved in making policy regarding CHWs at the state and national levels.

The phase of the CI Project covered by this manuscript occurred in the midst of at least four cataclysmic events which strongly influenced the project. These included the emergence of the COVID-19 pandemic; the uprising for racial justice that followed the police killings of George Floyd, Breanna Taylor, and Tony McDade; the largest economic crisis since the Great Depression; and an upsurge in white supremacist violence. As a project committed to health justice, the CI Project sought to respond to these crises in a variety of ways, some of which are detailed below.

At the beginning of the period described in this manuscript, there was one CHW on the five-person Leadership Team, and multiple CHWs in the Advisory Group. At the end of this period, the Leadership Team comprised three CHWs and three non-CHW collaborators, and a CHW Council consisting of four CHW leaders had been formed through a national recruitment process.

The CI Project uses popular education as a theoretical framework, an organizing philosophy, and an educational methodology. Also referred to as “people's education,” popular education creates settings in which people most affected by inequities can share what they know, learn from others in their community, and use their knowledge to create a more just and equitable society (16). Popular education and the CHW model grew out of many of the same historical roots and share key principles, such as the ideas that people most affected by inequity are the experts about their own lives, and that experiential knowledge is just as important as (and sometimes more important than) academic knowledge (17).

### Collaboration With the CDC

This project benefited from the close collaboration of the CHW Work Group at CDC (the Work Group). The Work Group first convened in 2011 as an informal multidisciplinary group, composed of volunteers from CDC's National Center for Chronic Disease Prevention and Health Promotion. Since then, the Work Group has expanded to include representatives from across CDC. The mission of the Work Group is to facilitate, support, and advance CHW initiatives and policies to help accomplish public health goals.

The 2019–2020 CI work plan was created jointly by the CI Project Leadership Team and colleagues at CDC and NACDD. CDC and NACDD colleagues provided input into two major aspects of the Project: the choice of priority indicators and the development of indicator profiles. CDC colleagues prioritized development of indicators for policy and systems change at the program and state levels, since they had identified this as a gap in previous work and it is central to CDC's CHW Sustainability Strategy (18). The other constructs prioritized by CDC colleagues included: *Participant Health and Social Needs*; *Participant Self-Reported Health Status*; *CHW-Facilitated Referrals*; and *CHW Integration into Teams*.

The CI Leadership Team prioritized five constructs that had been highlighted by stakeholders: *CHW Compensation, Benefits, and Promotion*; *CHW Enactment of the 10 Core Roles* (as identified in the CHW Core Consensus or C3 Project) (19); *CHW Involvement in Policy Making*; *Participant Empowerment*; and *Participant Social Support*.

CDC colleagues were involved in the development of profiles (detailed documents including a definition of the construct, purpose and rationale of the indicator, a description of the indicator, recommendations for how to operationalize it, and other information) for the 10 priority constructs. CDC and NACDD colleagues also helped to develop criteria to identify and select key stakeholders who would provide feedback for the content and operationalization of the indicators. They participated actively in bi-monthly Advisory Group meetings and the 2020 Summit (see below) and met with the Leadership Team to discuss the dissemination plan.

## Key Programmatic Elements

The collaborative methods used in this project included a review of peer-reviewed and non-peer reviewed literature, and comprehensive stakeholder engagement culminating in a 1.5-day online Summit held in 2020. Findings from all methods were triangulated to produce results and identify lessons learned. Both the findings of this project and popular education suggest that equitable engagement of marginalized individuals and communities including CHWs depends on thoughtful and diligent work before, during and after engagement opportunities. For this reason, the processes used and how they were influenced by popular education are described in some detail.

### Literature Review

A review of peer-reviewed and non-peer reviewed literature about measurement of the 10 priority constructs was conducted to identify existing measurement approaches and promising paths forward for indicator development and validation. The Leadership Team divided the 10 priority constructs among four members of the team (including one CHW), and each member undertook the literature reviews for their respective constructs, consulting each other as needed.

It was difficult to conduct a truly systematic literature review across all 10 priority indicators. Leadership Team members were tasked with reviewing literature for a total of 10 constructs, some of which have been studied for decades. In some cases, Leadership Team members were able to build on literature reviews they had begun as much as 20 years earlier, for constructs that have been well-defined (e.g., empowerment and social support). In these cases, they used academic databases such as EBSCO host to update searches in other databases including Academic Search Complete, Academic Search Premier, E-Journals, Health Source: Nursing/Academic Edition, *Fuente Académica*, MasterFILE Premier, MedicLatina, Medline, and Psychology and Behavioral Sciences Index.

For the self-reported health status construct, an initial search on general health-related quality of life revealed two widely accepted measures: the SF-12 (20) and the CDC Healthy Days measure. Once these measures had been identified, a search was conducted in PubMed, filtering for publications in the U.S. from 2010 to 2020. For other well-defined constructs (i.e., teamness, a construct contained within *CHW Integration into Teams*), research reviews exist and were consulted.

In other cases (notably *CHW Enactment of the 10 Core Roles, Policy and Systems Change*, and *CHW Compensation, Benefits, and Advancement*), reviewers were unable to identify peer-reviewed literature. Leadership Team members used their networks and general abilities to search for various kinds of literature to conduct what they felt were sufficiently thorough reviews of both peer-reviewed and non-peer-reviewed literature. They consulted CDC documents, reports, presentations, and parallel literature in other fields and/or current practice in the CHW field.

Some indicators (e.g., *Participant Health and Social Needs*), while not well-defined as constructs, are frequently measured in the CHW field, and Leadership Team members were able to base indicators on existing, widely used measures. This was also the case with *CHW-Facilitated Referrals*, where activity tracking forms and individual patient self-management assessments from programs in Oregon and Michigan were used. Reference lists from journal articles were also searched. The most useful tools were derived from other organizations and measurement efforts.

While most of the literature review occurred before the formal stakeholder engagement described in the next section, stakeholders including CHWs have been engaged in choosing and defining constructs and identifying potential indicators since the CI Project began. Stakeholders provided input into the project at multiple times and venues, including the organizing Summit in 2015; 2016 and 2019 pre-conference workshops at the Annual Meeting of the American Public Health Association (APHA); multiple interactive workshops at state and local conferences; and bi-monthly Advisory Group calls. Since 2015, a substantial portion of several Advisory Group calls has been dedicated to specific constructs, eliciting how Advisory Group members have defined and measured the construct in their own programs and settings.

### Stakeholder Engagement

A formal process of stakeholder engagement, emphasizing engagement of CHWs, was the centerpiece of the 2019–2020 scope of work. Input was sought from stakeholders at various times, in various venues, and on various questions about both overarching issues and specific constructs and indicators.

#### APHA Pre-conference Workshop, November 2019

In November 2019, the Leadership Team held a 2.5 h by-invitation workshop at the APHA Annual Meeting. Invitees included all current members of the Advisory Group as well as key partners from CDC and the National Association of Community Health Workers (NACHW). The primary goal of the workshop was to invite feedback on: (1) the tentative list of 10 priority constructs, (2) the stakeholders who would help guide indicator development for these constructs, and (3) the methods for engaging these stakeholders. The Leadership Team also aimed to develop community through face-to-face interaction. Eighteen people participated in the workshop, including CHWs, supervisors, researchers/evaluators, program directors, and others.

After a welcome and opening dinámica, facilitators reviewed objectives and action steps for the 2019–2020 work plan. They then divided participants into cooperative learning groups and elicited feedback on the criteria for choosing stakeholders, the initial list of stakeholders, and the proposed list of priority constructs. The workshop concluded with a large group report back, a brainstorm of next steps, and a group evaluation of the meeting.

#### Regular Meetings of the Project Advisory Group

Since the organizing Summit in 2015, the Advisory Group has met monthly or bi-monthly. The Advisory Group distribution list has grown from 16 to 170+ individuals from 30 states and the District of Columbia. Much of this growth occurred during 2019-2020, thanks at least in part to funding from the CDC. Many regular Advisory Group participants are active members of their state's or region's CHW association or network. Multiple researchers and CHW program evaluators also regularly attend. Attendance at meetings has climbed steadily from ~10 to more than 50 attendees.

Advisory Group meetings use popular education methodology and building community is the first objective of every meeting. When participant numbers allowed, facilitators set aside time at the beginning of every meeting for all participants to introduce themselves. Recently, facilitators have begun meetings with short breakout groups to further relationship building. Meetings always include an update from the Leadership Team members, who rotate facilitation responsibilities. In addition to focused discussion on specific indicators or topics, facilitators ensure time for participants to provide meaningful feedback on the information shared.

#### Individual Interviews and Focus Groups

Before the COVID-19 pandemic, the Leadership Team developed a plan for obtaining stakeholder feedback on the priority indicators that relied heavily on volunteers from the Advisory Group conducting in-person focus groups with CHWs and other stakeholders in their area. The Leadership Team developed a detailed lesson plan, PowerPoint, note-taking template and indicator grid (Table 2), and provided guidance to volunteers on how to use the materials. However, due to the pandemic, only two in-person focus groups took place. As the breadth of the pandemic became clear, the Leadership Team adapted the plan to rely on remote, web-based focus groups and one-on-one interviews. Ultimately, five focus groups were conducted with CHWs and other staff representing a state CHW association, a community-based organization, a state health department, the CI Advisory Group, and the research and outcomes arm of an urban health institute.

**Table 2:** Indicator grid.

**Construct**	**Definition**	**Rationale for measuring**	**How to operationalize**
#1CHWs' level of compensation, benefits, and promotion (PROCESS)	The salary paid to CHWs in relation to their FTE and local cost of living, in addition to the presence or absence of various benefits, as well as opportunities for promotion	*Justice*: Insufficient payment is exploitative and unfair. (2) *Effectiveness/performance*: Sufficient compensation allows CHWs to dedicate their full time and attention to community health work because it provides for all their material needs. (3) *Addressing poverty and lack of good jobs within communities*: Sufficient compensation for CHWs can facilitate a pathway out of poverty over the long-term. Living wage CHW jobs provide job development in communities.	Method 1: CHW surveys Method 2: CHW employer surveys
#2CHW enactment of the 10 core roles (PROCESS)	How often individual CHWs or a group of CHWs within a program, organization, state, or region enacts each of the 10 core roles defined by the CHW Core Consensus (C3) project.	Collecting these data is critical to evaluating the unique contributions of CHWs and the outcomes they achieve. Research suggests that CHWs are better able to contribute to improving health and decreasing health inequities when they are supported to play a full range of roles. In addition, clarity about CHW roles can foster CHW integration into teams and will also allow training to be geared to meet CHWs' needs, and/or to emphasize the necessity of playing a full range of roles.	CHW Encounter Forms or other forms used to track CHW interactions with individuals and groups.
#3CHW-facilitated referrals (PROCESS)	Completed referrals facilitated by the CHW, through which the participant successfully receives attention, care, and/or resources from a clinic, other healthcare or social service agency or public service.	Making and facilitating referrals for community members to needed and appropriate health or social services is directly connected to at least 7 of the 10 core roles of a CHW as defined by the C3 project. This key component of CHW work is currently being measured at the individual programmatic level, and although there are various models and survey questions used within the domestic and international setting, there is no recommended standard instrument that can be used to generate national data sets for this activity.	CHW Encounter Forms or other forms used to track CHW interactions with individuals and groups (paper or digital).
#4CHWs' involvement in decision- and policy-making(PROCESS)	The extent to which a CHW is able to be involved in policy making both within their own organization and in the larger community on work time and/or as part of their volunteer commitment.	Policy making is one of the three core functions of public health. CHWs' ability to address the social determinants of health and eliminate health inequities depends on their ability to create and influence health-promoting policy, both within and outside their employing agency. Being able to influence policy depends on knowing who to work with, being trusted by other policy actors, and being supported to engage in policy making on work time.	CHW surveys
#5Extent to which CHWs are integrated into teams (for example, health care teams) (PROCESS)	The extent to which CHWs are members of a collaborative and communicative “team” with other providers (i.e., nurses, doctors, social workers, health educators, pharmacists, etc.) within a clinic, school, social service agency, etc.	Well-functioning, transdisciplinary teams have been recognized by the Institute of Medicine as key to the safety and quality of care across multiple settings. Integration of CHWs into transdisciplinary healthcare and social service teams is widely recognized as key to the effectiveness, cultural appropriateness, and quality of care. Despite wide recognition of its importance, integration of CHWs into care teams and its impact on team functioning are rarely measured. Also, while care teams more frequently include CHWs, this often may not yet represent their meaningful integration as full participants in care teams.	CHW surveys
#6Participant self-reported physical, mental, and emotional health (OUTCOME)	The self-reported assessment of perceived physical, mental and emotional health and quality of life.	An indicator of self-reported health is important for monitoring and assessing the perceived general and functional health and quality of life of individuals and populations. It is widely used in the U.S. and worldwide, relatively easy to measure, and generally correlates well with clinically measured health status, use of health services and health care costs. Self-reported health “incorporates the voices of individuals” and provides “a more holistic view of overall health.”	Participant surveys
#7Participant health care and social needs (OUTCOME)	Health care and social needs currently experienced by the participant.	A key proven outcome of CHW action is more secure access among participants (and their households) to primary care and various social services that may be needed (e.g., food banks, housing support, legal support, etc.). More secure access to primary health care and social services, in turn, is crucial to the well-being of marginalized households and communities.	Participant surveys or assessments
#8Participant social support (OUTCOME)	The level of support (i.e., assistance/help) that participants perceive from others to deal with regular and emergent life challenges, including economic, social, health, and emotional challenges.	The presence of social support has been associated with faster recovery from illness, responsiveness to treatment in stress-related illnesses and fewer pregnancy complications, and decreased levels of depression, greater life satisfaction, and better well-being. Lack of support is strongly associated with increased morbidity and mortality. CHWs provide social support both directly, by accompanying community members, and indirectly, by linking them to existing groups and starting new ones.	Participant surveys
#9Participant empowerment (OUTCOME)	A composite measure assessing both actual and perceived empowerment. Includes the following domains: self-efficacy, sense of community, perceived control at the community level, decision-making ability, education/knowledge/skills, critical consciousness, optimism, inner peace, communication, resources.	Empowerment is “recognized by the World Health Organization and health agencies around the world as a core concept in health promotion and integral to the achievement of social equity.” Empowerment independently predicts self-reported health status and depression, and is in the pathway to improved health, making it a good intermediate measure of health status. Increasing empowerment is seen as a critical CHW function; it has also been hypothesized that CHWs are unique among other health and social service professionals in their ability to support participants to increase their empowerment.	Participant surveys
#10Policy and system change: program/employer level (OUTCOME)	Policies and system changes that address CHW workforce development and sustainability. For our 2019–2020 work, we focused on policies related to CHW workforce development (training, payment, etc.).	The CHW workforce is best respected and stabilized through policies that support their sustainability, including a recognized definition and scope of practice/roles, core-competency-based training, voluntary certification mechanisms, appropriate supervision, and payment mechanisms that support sustained employment, e.g., general funds and insurance company payment. CHW employers and programs can institute these policies at the CHW employer/program level.	CHW program/employer surveys
#11Policy and system change: state level (OUTCOME)	(see above)	The CHW workforce is best respected and stabilized through policies that support its sustainability and integrity, including a recognized definition and scope of practice/roles, core-competency-based training, voluntary certification mechanisms, appropriate supervision, and payment mechanisms that support sustained employment, e.g., general funds and insurance company payment (CDC, May 2019). State governments can facilitate policy and systems changes that support CHW programs, employers and the CHW workforce.	Surveys of a state government's policies and practices

*The indicators proposed below rest on the following set of assumptions:*

*1. CHWs^2^ will be responsible for (i.e., involved in) collecting the data for many of these indicators*.

*This is true, for example, of indicators that are included in pre-post surveys/assessments with participants*.

*2. When they are fully disseminated for use to programs, the indicators will be accompanied by a manual that will include further explanation of the meaning and intent of each indicator, so that those who collect the data are able to interpret them in culturally-centered ways*.

*3. We are proposing quantitative indicators because they are easiest to implement in a consistent and reliable way. We recommend that these indicators be used along with qualitative methods that are specific to the culture/community and setting*.

*4. Whenever possible, we recommend that indicators be operationalized in existing data collection and/or case management tools, to reduce the burden on CHWs and data management staff*.

*5. When we recommend an indicator be collected on a CHW Encounter Form, that can occur either on paper or via an online case management database like RedCap, CareScope, ETO, SMART Sheets, etc*.

*6. Assessing CHWs' contributions to improving population health (e.g., with community-level indicators) is crucial. However, it is beyond the scope of most or all CHW programs to do that on their own; for this reason, among others, we are not recommending community-level indicators. We are, however, recommending collection of a participant general health indicator (Indicator #6, below)*.

*7. Many things are beyond the immediate control of the CI Project, such as the multiple titles used for CHWs. However, if we collect these data systematically, some things should become more consistent, such as CHW job descriptions that are based on the APHA definition and the 10 core roles as identified in the C3 Project*.

*8. For collecting initial assessment data, some CHW programs use Intake Forms, some use a pre-assessment, and some use both. Any of the participant outcome indicators that we recommend for inclusion in a pre-assessment could also be included in an Intake Form, as long as that same indicator is repeated at regular intervals to assess change*.

*9. Along with assessment and assurance, **policy development** is one of the three core functions of public health (https://www.cdc.gov/nceh/ehs/10-essential-services/resources.html). As essential public health professionals, Community Health Workers also engage with their communities in developing policies that promote health, prevent disease, and ameliorate existing health inequities*.

*10. We acknowledge the importance of health care utilization and cost measures; however, it is impossible to create or identify one utilization measure that will work in all cases, especially because not all CHW programs have access to this data*.

**Figure 1:**
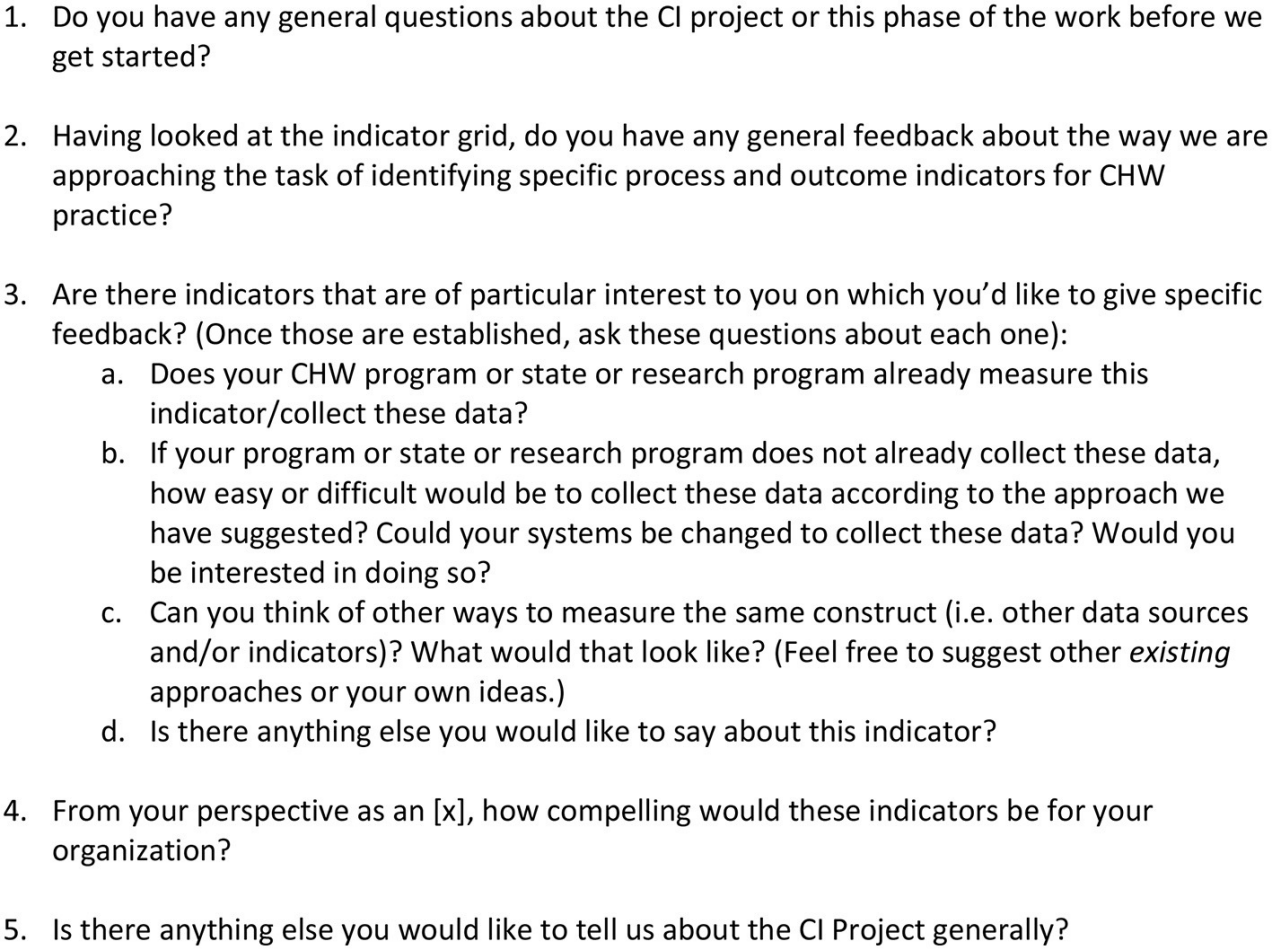
Individual interview guide.

Leadership Team members also conducted seven individual interviews, as well as several informal conversations using an interview guide (see Figure 1). Combining focus groups and one-on-one interviews, 46 people were reached. While the Leadership Team had hoped to reach more people, they were able to reach most major stakeholder groups. By the end of the process, based on iterative analysis after each interview and conversation, Leadership Team members felt they had reached saturation on several questions and concepts, providing confidence in the findings.

Early in the development of the Stakeholder Engagement Plan, the Leadership Team developed a system for analyzing the feedback received. This plan consisted of at least one Leadership Team member (and sometimes two) doing a line-by-line analysis of the transcript and/or notes taken by the facilitators and interviewers. (Some interviews and focus groups were audio recorded; during others, facilitators took careful notes.) Subsequently, summaries of the feedback on each indicator were created and a list of cross-cutting themes (identified in the Results section below) was compiled and added to the previously developed indicator grid (Table 2). This allowed the Leadership Team member responsible for developing each indicator to quickly see the individual feedback as well as the cross-cutting themes, which were identified through a process that combined both inductive and deductive coding (21, 22). Leadership Team members used these materials to discuss and make changes to the indicators and resulting grid.

#### Online Summit, May 2020

The second CI Summit was the major culminating stakeholder engagement activity to solidify the indicators and identify next steps for the Project. The Leadership Team began planning in January of 2020 and moved toward inviting participants to a 2-day Summit in Portland, Oregon. As the severity of the COVID-19 pandemic became increasingly clear, the Leadership Team made iterative shifts in the planning, moving first to the idea of a hybrid Summit, and then, by March 20, deciding to conduct the Summit entirely online. The Summit took place on May 14-15, 2020.

The Leadership Team sought to invite a diverse group of ~20 CHWs, researcher/evaluators, CHW program staff, health system staff, state and local health department staff, and colleagues from CDC and NACDD. Initial invitations were sent in early March, when an in-person gathering was still planned. This led to an overrepresentation of people from the West Coast, for whom travel costs would have been lower. Once organizers decided to make the Summit entirely virtual, they expanded the group to include more people from other parts of the country. A total of 39 people (including facilitators and staff from NACDD and CDC) participated in the Summit, of whom 16 identified as CHWs. Further information about planning for the Summit and how popular education was used in the online environment, as well as a final report from the Summit are available on the CI Project webpage (23).

A systematic approach was used to document the process and outcomes of the Summit. First, a 21-page document that included notes from all the plenary sessions at the Summit was prepared. In addition, 10 individual documents with notes on each indicator and three individual documents with notes on the piloting process were created. All these documents informed the Indicator Profiles. Finally, a checklist of important considerations for the piloting process was developed.

## Findings and Discussion

### Literature Review

A primary finding from the literature review was that there was a great deal of literature about some constructs and a paucity of literature about other constructs. While some constructs had not been measured in CHW programs, they had been measured in other settings. For some of the indicators, it made sense to adopt and adapt published and validated scales, whereas for other indicators, there were no published, validated measurement approaches. Thus, indicators had to be developed “from the ground up,” by proposing questions/items that have been used in CHW program evaluations but not necessarily published and validated, and/or by developing brand new questions/items that were suggested and endorsed by our stakeholders. Another general finding was that some construct names needed to be changed to align with what is used in peer-reviewed literature and in community health practice. This was the case with *Participant Health and Social Needs* (which was originally titled, *Participant Access to Health and Social Services*).

Another important finding from the literature review was that a project with similar goals to the CHW Common Indicators Project—yet focused on low- and middle-income countries rather than the United States—had recently been conducted (24). The existence of this project, known as the Frontline Health Project and funded by the Bill and Melinda Gates Foundation, further confirmed the timeliness and potentially global significance of the CHW Common Indicators Project. There was a great deal of overlap between the two projects in overall measurement frameworks and in respective lists of recommended evaluation constructs. In this case, the literature review informed the stakeholder engagement by prompting the Leadership Team to engage leaders within the Frontline Health Project, who provided input on the CI Project indicators, shared resources, and joined the Advisory Group. One important distinction is that while the CI Project has focused on engaging CHWs as leaders in the work, publications by the Frontline Health Project do not provide evidence of such engagement. This likely leads to important differences between the projects in overall measurement frameworks and recommended constructs and indicators, a review of which is beyond the scope of this article.

### Stakeholder Engagement

The most important findings from the stakeholder engagement are reflected in Table 2. Specific findings from specific stakeholder engagement activities are highlighted below.

#### 2019 APHA Pre-conference Workshop

Participants in the workshop endorsed the tentative list of 10 priority constructs and strongly endorsed selected constructs including *Participant Empowerment, CHW Integration into Teams*, and *CHW Compensation, Benefits, and Promotion*. In addition, they urged Leadership Team members to make important additions to the stakeholder list, including CHW employers, payers/health plans, FQHCs, and state health department representatives. They identified a need to clarify and further develop the *CHW-Facilitated Referrals* construct, so as not to make CHWs responsible for “failed” referrals (when, for example, services don't exist and/or are substandard, not culturally competent, inaccessible, etc.). Finally, they identified a need to acknowledge the importance of health care utilization and cost measures but make clear it is impossible to create one utilization/cost measure that will work in all settings.

#### Advisory Group Meetings

In the meeting evaluation, participants regularly provide useful corrective feedback, for example: “Sometimes we need a space to discuss things that aren't on the agenda, so we have some time to discuss and organize.” They also frequently express appreciation for the way meetings are conducted, such as the following from the May 2020 meeting: “As usual, I appreciate the level of organization that gets us through a lot of stuff. I continue to be very excited about sitting on this group.” The trust and community building processes inherent in the Advisory Group meetings and other CI Project activities have been essential to the broad consensus and growing, nationwide uptake of common indicators for evaluating CHW practice.

#### Focus Groups, Individual Interviews, and May 2020 Summit

Overall, this phase of the Project demonstrated the broad acceptability of the measurement framework developed within the CI Project. That framework centers CHWs' 10 core roles with an explicit goal to ensure that all 10 roles are understood and practiced within CHW programs. The framework also highlights key kinds of support that CHWs need to be successful in all 10 roles, and outcomes that CHWs are particularly capable of bringing about—not only at the level of individual participants in CHW programs (e.g., wellbeing and social support), but also at the level of policy and systems change to address structural determinants of health inequities. Notably, the framework defines and forefronts a multi-level indicator of empowerment, which both the literature and our experience suggest is among the most significant and emblematic outcomes of CHW work. This framework reflects the deep participation and leadership of CHWs within the Project, who aim to protect the integrity and advance the self-determination of the workforce. Non-CHW stakeholders generally affirmed the importance of these key features of the measurement framework, as well as the principle that CHWs must be deeply involved in evaluating their own profession.

Generally, stakeholders expressed enthusiasm and approval for the choice of indicators as well as the proposal to operationalize the indicators in existing tools such as encounter forms, CHW surveys, CHW employer surveys, participant surveys, and state performance reports. Stakeholders reinforced the importance of complementing quantitative indicators with qualitative, narrative, and ethnographic assessment methods. They pointed out that narrative methods and storytelling are uniquely powerful and effective ways to document how and why CHW practice is effective in various cultural settings, and to clarify the kinds of changes that are necessary to improve systems, provide adequate support to CHWs and their communities, and achieve health equity.

## Lessons Learned

An important lesson reaffirmed during this phase of the CI Project was that obtaining meaningful input from a diverse group of stakeholders about a project primarily concerned with measurement and evaluation in the midst of a pandemic is challenging and requires thoughtful planning, skillful use of popular education methodology, and a team that is aligned around common goals and principles.

During the early stakeholder focus groups, it was found that many stakeholders who were not trained as evaluators commented not on how to *measure* concepts like social support, but rather how to *increase* social support among program participants and what they need to be successful in their work. Making the distinction between *doing the work* and *measuring the work* was challenging for volunteer focus group facilitators. This was partly because most CHW program staff are focused on doing, not measuring, their work and CHWs remain largely marginalized from evaluation and research processes.

Similarly, based on the summaries of indicator-specific input created after the Summit, it was clear that the way the indicators were explained by the small group facilitators affected the feedback group members provided on those indicators. As the CI Project moves to piloting, it will be important to assure that indicators are explained in a consistent way.

Interestingly, many stakeholders, including CHWs, resisted shortening the length of (i.e., reducing the number of items within) the indicators, even when the goal of keeping the indicators relatively quick and easy to use had been emphasized. This was generally because stakeholders felt that cutting proposed items would eliminate important aspects of CHW practice and participant outcomes. At the same time, stakeholders readily appreciated that program capacity must be developed to *measure the work*, so that measurement and evaluation help rather than hinder CHWs in *doing the work*. As we move forward with the piloting process, it will therefore be important to carefully communicate with CHWs and other stakeholders the pros and cons of shorter surveys, and the fact that funding is necessary to pay for CHWs' and others' time involved in new data collection and reporting. This is a reflection of how the goals of the CHW Common Indicators Project are intricately tied to the issue of securing sustained funding for CHW programs generally.

Other lessons learned included the importance of clearly communicating project assumptions at the outset of any engagement activities. Specifically, stakeholders expressed the importance of making it clear that:

- the indicators will be accompanied by a manual that will carefully explain the meaning and intent of each indicator;
- the Project will recommend that indicators be operationalized using existing data collection and/or case management tools, whenever possible, to reduce the burden on CHWs and other data management staff;
- quantitative indicators are proposed because they are easiest to implement in a consistent and reliable way, and not because they are of higher value than qualitative methods;
- health care utilization and cost measures are important but not included, because it is impossible to create one utilization or cost measure that will work in all cases and not all CHW programs have access to this data; and
- while the CI Project is unable to control the fact that CHWs are often given multiple different titles, the project's recommended indicators can help bring about more consistency in CHW job descriptions, given the indicators' built-in emphasis on the APHA definition of a CHW as well as the C3 Project's definition of the 10 core roles of CHWs (19).

Though it was enforced by the pandemic, heavier reliance on individual interviews was beneficial to the goal of collecting constructive feedback on the proposed and evolving indicators. The experience doing focus groups revealed that the kind of input needed was easier to obtain one-on-one.

## Future Applications

With assistance from colleagues at CDC and NACDD and participants in the 2020 Summit, the Leadership Team has identified several next steps, in which the CI Project is currently engaged. The first step is piloting the indicators developed during 2019-2020 and developing a manual/toolkit with information crucial to piloting, including definitions, intent, background, and methods of calculation. A participatory and developmental evaluation for the pilot will also be conducted, with pilot sites actively involved in the on-going development of the evaluation plan. Other next steps include developing an indicator for reflective supervision, continuing to build project infrastructure, and strengthening the CI Project's CHW-led and community-based methodology. In pursuit of the final goal, following a national recruitment process, in August of 2020 the CI Leadership Team expanded and is now 50% CHW and 50% CHW ally. In addition, the team created a CHW Council composed of four CHWs with experience in research and evaluation. Finally, the Leadership Team chose and adapted a racial equity tool to guide future project decisions.

## Conceptual or Methodological Strengths and Constraints

This phase of the CI Project had several strengths, including a well-developed network of 180+ individuals who possess a variety of skills, perspectives, and knowledge based on lived experience and are committed to improving measurement in CHW programs; and the Leadership Team's dedication to and capacity in using popular education methodology. This methodology allows the Leadership Team to operationalize our commitments to community-based participatory research; shared power; racial, social, and health justice; and a non-hierarchical approach. All members of the Leadership Team are deeply committed to the project, and possess a well-rounded set of skills as CHWs, researcher/evaluators, and program managers. An additional strength is our strong relationship with NACHW and various state CHW organizations. The CI Project benefited from the dedication and knowledge of the CDC CHW Work Group members and partners at NACDD. Also, using an online platform for the 2020 Summit facilitated inclusion of a larger and more diverse number of stakeholders.

The work discussed in this article had several constraints and limitations. A major limitation was the COVID-19 pandemic, which required project partners to be creative in how they collected stakeholder feedback and built community. Funding constraints and lack of full-time staff on the project limited the number of hours staff could dedicate to various project activities. Despite attempts to mitigate its effects, limited racial/ethnic diversity on the Leadership Team, the fact that all three members with doctoral degrees were white, and that only one member was a CHW unquestionably influenced power dynamics and meant that the Leadership Team lacked crucial perspectives. Recognizing this led to an intentional process of increasing both racial/ethnic diversity and CHW representation on the Leadership Team and creating a CHW Council to provide additional CHW input into decision-making.

## Conclusion

The CDC-funded 2019-2020 work plan was pivotal for the CI Project. Having funds to pay salary and stipends allowed Leadership Team members and Summit facilitators to set aside time to focus on this project, leading to substantial progress on the overall development of the CI Project. With the participation of multiple stakeholders, particularly CHWs, 11 profiles were developed for 10 priority constructs. The profiles include information about why the indicators are important, why they should be measured in particular ways, and how data should be calculated. In addition, largely based on the success of the Summit, the CI Project substantially expanded the constituency committed to the Project. In 2020–2021, Leadership Team members look forward to piloting the indicators, developing materials and methods to support the piloting, strengthening the work through the application of an explicit racial equity lens, and continuing to expand CHW involvement and capacity as the researchers and evaluators for and about their field.

Some persistent and important questions remain. The CI Project is premised partly on the idea that policy makers and funders require additional data about CHW outcomes before they will agree to sustainably finance CHW programs. Yet the same does not seem to be true of professions and programs staffed by more privileged people. They do not have to produce data about their value, at least not in the same way. The CI Project will continue to problematize that fundamental issue while continuing to develop indicators, based on the conviction that if CHWs and dedicated allies do not do so, someone else will, with potentially dangerous consequences for CHW autonomy and self-determination.

## Data Availability Statement

The original contributions presented in the study are included in the article/supplementary material, further inquiries can be directed to the corresponding author/s.

## Author Contributions

KR and NW contributed equally to the work plan and writing of this manuscript. KM contributed to the work plan and writing of this manuscript. TC-D contributed to the work plan and reviewing of this manuscript. VA, PJ, and SM-J contributed to the review of this manuscript. All authors contributed to the article and approved the submitted version.

## Conflict of Interest

NW was employed by Wiggins Health Consulting, LLC. The remaining authors declare that the research was conducted in the absence of any commercial or financial relationships that could be construed as a potential conflict of interest.

## Footnotes

^1^This publication was supported in part by Federal Award Number: 5-NU38OT000286-02, CFDA Number: 93.421, from the Center for Disease Control and Prevention, via the National Association of Chronic Disease Directors.

^2^Please note that in the CHW Common Indicators Project, the term “Community Health Workers” (CHWs) is inclusive of Promotores/as de Salud and Community Health Representatives.
